# Baclofen in gamma-hydroxybutyrate withdrawal: patterns of use and online availability

**DOI:** 10.1007/s00228-017-2387-z

**Published:** 2017-12-03

**Authors:** Christopher N. Floyd, David M. Wood, Paul I. Dargan

**Affiliations:** 1 Clinical Toxicology, Guy’s and St Thomas’ NHS Foundation Trust and King’s Health Partners, London, UK; 2 Faculty of Life Sciences and Medicine, King’s College London, London, UK

**Keywords:** Pharmacotherapy, Drug abuse, Clinical toxicology, GABA

## Abstract

**Purpose:**

Gamma-hydroxybutyrate (GHB) withdrawal is a life-threatening condition that does not always respond to standard treatment with benzodiazepines. Baclofen has potential utility as a pharmacological adjunct and anecdotal reports suggest that it is being used by drug users to self-manage GHB withdrawal symptoms. Here, we investigate current patterns of use and the online availably of baclofen.

**Methods:**

Data triangulation techniques were applied to published scientific literature and publicly accessible Internet resources (grey literature) to assess the use of baclofen in GHB withdrawal. An Internet snapshot survey was performed to identify the availability of baclofen for online purchase and the compliance of retailers with the UK regulations. Data were collected according to pre-defined criteria.

**Results:**

A total of 37 cases of baclofen use in GHB withdrawal were identified in the scientific literature, as well as 51 relevant discussion threads across eight Internet forums in the grey literature. Baclofen was available to purchase from 38 online pharmacies, of which only one conformed to the UK regulations.

**Conclusions:**

There is limited published evidence on the use of baclofen in GHB withdrawal, but both scientific and grey literature suggests clinical utility. Online pharmacies are readily offering prescription-only-medication without prescription and due to inadequate regulation, pose a danger to the public.

**Electronic supplementary material:**

The online version of this article (10.1007/s00228-017-2387-z) contains supplementary material, which is available to authorized users.

## Introduction

Gamma-hydroxybutyrate (GHB) is a naturally occurring metabolite of the neurotransmitter gamma-aminobutyric acid (GABA) and was first synthesised as an anaesthetic agent in 1964 [1]. A high incidence of adverse effects during early clinical trials restricted its use in anaesthesia, but at lower doses, it has found some clinically utility and is licenced for treating sleep disorders (particularly narcolepsy with cataplexy) and alcohol dependence [2]. In addition, it has been used illicitly in both bodybuilding and as a recreational drug [3, 4].

Use of GHB and its precursors (gamma-butryolactone (GBL) and 1,4-butanediol (1,4-BD)) have a dose-dependent effect on glutamate and dopamine release. At low dose, stimulation of GHB receptors increases dopamine release resulting in stimulant-like effects, whilst at higher doses, binding to GABA-B receptors results in hypnotic effects and eventually coma [5]. There is no specific antidote for GHB and so acute toxicity is managed by supportive care which, in the case of significant consumption, may include endotracheal intubation for airway protection [6].

The first reports of physical dependence and consequent withdrawal syndrome were the result of both supervised clinical and unsupervised recreational use [7–9]. Dependence results from modulation of baseline neuronal excitability in a mechanism that is analogous to that seen with alcohol and benzodiazepines dependence [10]. A terminal half-life of approximately 40 min means that those with dependency need to dose GHB/GBL every few hours [11], and interruption to this schedule results in a withdrawal syndrome typified by sympathetic over activity, florid delirium and profound neuropsychiatric features [6, 12]. Standard treatment involves high dose benzodiazepines (often up to 200 mg/day diazepam equivalent) with the possible addition of a range of other drugs including antipyschotics, barbiturates and anticonvulsants [13]. Benzodiazepines however are positive allosteric modulators of GABA-A receptors rather than GABA-B where GHB acts, and so despite their action to maintain GABAergic stimulus and prevent harmful neuronal excitability, they may not be the ideal pharmacotherapy [14]. It has therefore been suggested that the use of specific GABA-B agonists such as baclofen may provide clinical benefit in the management of withdrawal beyond that offered by benzodiazepines [6].

Baclofen is a direct agonist at GABA-B receptors [15] and has been widely used for the control of spasticity since the 1960s, where it acts to inhibit spinal afferent pathways and reduce motor neurone activity [16]. Animal work in both small mammals and primates has demonstrated that chronic GHB administration induces cross tolerance to baclofen [17, 18], and one case report described the use of baclofen as rescue therapy when increasing benzodiazepine dose failed to control GHB withdrawal [19].

Anecdotal reports from inpatients treated for GHB/GBL dependence and withdrawal describe dependent users purchasing baclofen from the Internet to self-medicate. The primary aim of this work was to understand and catalogue the use of baclofen in the management of withdrawal from GHB and its precursors. Triangulation of data from published scientific literature and publicly accessible Internet resources has enabled us to address the following objectives: (i) review scientific literature for reports of baclofen use by healthcare professionals, (ii) review Internet forums for reports of baclofen use by the public and (iii) assess the availability of baclofen to purchase online.

## Methods

### Scientific literature

MEDLINE/PubMed was searched up until the 7th of February 2017 for all articles containing the keyword ‘baclofen’. The same keyword was used to search TOXLINE and EMBASE (up until the 12th of October 2017) and the abstracts of the European Association of Poisons Centres and Clinical Toxicologists (EAPCCT) and North American Congress of Clinical Toxicology (NACCT) congresses (from 2002 to 2016). All potentially eligible manuscripts were reviewed in full to identify those that investigated the use of baclofen in the treatment of GHB/GBL withdrawal. A secondary search was performed on the references of all eligible manuscripts to identify further relevant publications.

### Grey literature

Publicly accessible Internet resources were searched in March 2017 for descriptions of baclofen being used to manage GHB/GBL withdrawal. The search term ‘baclofen’ was entered into the Internet search engine google.co.uk with the search terms ‘GHB’, ‘GBL’ and ‘withdrawal’ added separately and in combination. Search results for each combination of search terms were reviewed sequentially until 20 successive irrelevant or duplicate websites were visited. The search terms were then entered into individual discussion forums and blogs identified through the search, as well as searching relevant forums and blogs which had been identified through previous work [20]. Reports of baclofen use to manage GHB/GBL withdrawal were analysed manually and data relating to dosing regimen and adjunct medication were recorded.

### Internet snapshot survey

An Internet snapshot survey for the availability of baclofen was undertaken on the 7th of February 2017 using methodology developed by the European Monitoring Centre for Drugs and Drug Addiction (EMCDDA) [21]. The term ‘buy baclofen’ was entered into the Internet search engine google.co.uk and approximately 603,000 results were returned. The first 100 search results were reviewed in full followed by sampling to exhaustion until 20 successive websites were defined as being irrelevant, unrelated or a duplication of sites already visited. No baclofen was purchased during this study, but the ability to proceed with an online purchase up to the point of entering financial and/or contact details was accepted as a successful transaction.

Data from each relevant website were recorded according to pre-specified criteria that covered both the pharmaceutical offering and details regarding the online pharmacy. Primary data recorded included (i) form(s) of baclofen available to purchase, (ii) cost, (iii) any requirement for a prescription, (iv) geographical location of the seller and (v) evidence of compliance with United Kingdom (UK) and European Union (EU) registration.

**Fig. 1:**
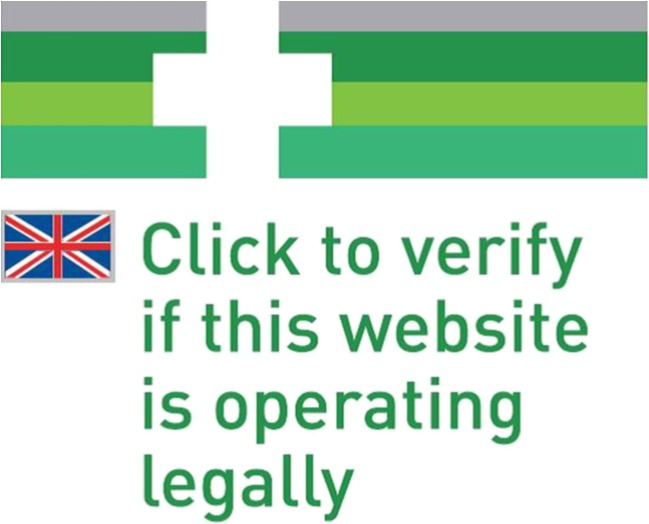
An example of the European Union common logo for UK-based online pharmacies

The cost of baclofen was recorded in British Pound Sterling (£) and where alternative currencies were displayed, the European Commission’s online conversion tool was used [22]. Geographical location was recorded according to the first-rank criterion of the provision of a bricks-and-mortar address; subsequently, any statement on the website indicating locations (such as reported adherence to particular laws and regulations) and finally, brand identity. Brand identity was defined as feature of a website that suggested geographical location but can be obtained without a physical presence (i.e. domain name, domain suffix, brand name or telephone number). To further attempt to identify location, a WHOIS query (a tool to find the name and location of the registered domain holder) was performed on each domain [23]. All UK-based online pharmacies are required to be registered with the Medicine and Healthcare products Regulatory Agency (MHRA), and listed on their ‘online medicines seller registry’ [24]. Similarly, all EU-based online pharmacies must be registered with their respective body and display the ‘EU common logo’ on their website (Fig. 1) [25]. The availability of an integrated language translation service, provision of information about baclofen, presence of consumer reviews for baclofen and option to buy in multiple currencies were also recorded.

### Ethical considerations

All information recorded from the Internet was obtained from ‘purely public sites’ without the requirement for secure or member access [26]. No identifiable information was recorded when analysing data from the grey literature search. This research was approved by the King’s College London Research Ethics Committee (reference RESCMR-16/17-2097).

## Results

### Published scientific literature

The literature search identified 4776 unique results and these are summarised in Supplementary Fig. 1. Five relevant publications were identified, consisting of which three were case reports and two clinical study protocols [19, 27–30]. Two further case reports were identified from abstracts of the NACCT [31, 32], as well as two case series from the Internet search [33, 34], and one case series from the secondary search of references from eligible manuscripts [35].

The identified cases (summarised in Table 1) discussed both the use of baclofen as an adjunct for the management of acute GHB withdrawal (*n* = 37) and the use of baclofen as maintenance therapy to prevent relapse (*n* = 12). The dose of oral baclofen used to manage withdrawal was 15–60 mg/24 h and in all cases, baclofen was combined with oral or intravenous benzodiazepines. In five cases, baclofen was commenced only when the existing pharmacological approach was felt to be unsuccessful, and the addition of baclofen was reported to be associated with a clinically significant improvement in all of the cases. Clinical outcomes of treatment were not universally reported, and other than one instance of deliberate overdose [36], there were no adverse events attributed to baclofen.

**Table 1:** Summary of case reports involving the use of baclofen in GHB withdrawal

Reference	Cohort details	Pharmacotherapy and outcome
McDaniel et al. 2001 [35]	Case series (*n* = 5)	All patients received benzodiazepines, with baclofen used in 3 cases; 5 mg TDS, 10 mg TDS and 5 mg QDS
	Inpatient	
	Multiple daily GHB/GBL doses	
		Other pharmacological adjuncts included gabapentin, trazodone, carbamazepine, clonidine, chloral hydrate, phenobarbital, valproate, bromocriptine and risperidone
Le Tourneau et al. 2008 [19]	Case report (*n* = 1)	Initiated on titrated GHB and lorazepam, but tonic-clonic seizures necessitated transfer to ICU
	Withdrawal from GHB (> 16 g/day) and benzodiazepines	
		Prescribed baclofen (5 mg then 10 mg TDS) with rapid improvement of symptoms
		Discharged on baclofen 10 mg TDS, and abstinent of GHB at 10 weeks.
Acciani et al. 2010 [31]	Case report (*n* = 1)	Lorazepam infusion titrated to 14 mg/h
	Inpatient	Addition of baclofen 10 mg TDS on day 3 led to immediate reduction in lorazepam requirement
	Hourly GHB/GBL use	
		Discharged on benzodiazepine taper; no relapse at 1 month
Bell et al. 2011 [27]	Case series (*n* = 19)	Initiated on hourly diazepam PRN ± baclofen titrated to 10 mg TDS. Baclofen was withheld if symptoms were adequately controlled with diazepam monotherapy (*n =* 1*)* or if patients became drowsy on diazepam monotherapy (*n* = 1).
	Mixed inpatient and outpatient	
	12–40 mL GBL per day	
		Sixteen patients completed withdrawal over 5–7 days
		No medications on discharge
		Fifteen patients remained non-dependent of GBL at 2 months
Wood et al. 2011 [34]	Case series (*n* = 8)	All patients received PRN benzodiazepines, with 4 given baclofen 10 mg TDS and 1 given baclofen 20 mg TDS
	Outpatient and inpatient	
	GHB/GBL use 1–4 hourly	
McDonough 2013 [33]	Case series (*n* = 8)	All received diazepam 10–20 mg TDS
	Inpatient	Four patients received baclofen (dose unclear)
	Mean 17 g/day GHB	
Colbyl et al. 2015 [32]	Case series (*n* = 6)	Two cases in ICU: Regular diazepam plus baclofen TDS (10–40 mg) and dexmedetomidine
	Inpatient	
	GBL use 2–4 hourly	Four cases: PRN diazepam, clonidine and baclofen 10-20 mg TDS
		Six cases discharged on baclofen taper
Kamal et al. 2015 [28]	Case series (*n* = 11)	Detoxification by titrating GHB dose and diazepam PRN
		Once abstinent of GHB, baclofen was commenced at 5 mg TDS and titrated to max. 60 mg/day
	Mixed inpatient and outpatient	
		Five patients remained abstinent during 12 weeks of baclofen treatment at a dose of 30–60 mg/day
	42–90 g/day GHB	

*GBL* gamma-butryolactone, *GHB* gamma-hydroxybutyrate, *ICU* intensive care unit, *PRN* as required, *QDS* four times daily, *TDS* three times daily

There are no published clinical trials into the efficacy of baclofen in the management and/or maintenance of GHB withdrawal, although our search strategy did identify two study protocols that are yet to report [29, 30].

### Grey literature (publicly accessible Internet resources)

#### Forums

The use of baclofen in GHB withdrawal was identified in threads across eight publicly accessible Internet forums [37–44]. A secondary search within these forums was possible without user registration in six instances, resulting in a total of 51 relevant threads in which the word ‘baclofen’ was mentioned in 138 separate posts. Seven of the forums provided timestamps on posts and the earliest identifiable mention of baclofen use in GHB withdrawal was 2008 [45].

Baclofen dose was mentioned in 16 threads, of which the majority consisted of users posting about their own withdrawal experiences rather than making recommendations for others to follow. The median 24 h baclofen dose was 105 mg (IQR 60–200 mg, range 25–4000 mg). The need to taper medication (and/or GHB/GBL) as part of the withdrawal process was a common theme, but only five provided any form of protocol suggesting how baclofen could be weaned [46–50]. Two of these protocols were the same, contained references to published scientific literature and were credited to NHS Lothian, although the origin of the protocols could not be confirmed [49, 50].

A total of 24 different drugs or drug classes were referred to as adjuncts to, or substitutions for, baclofen in GHB withdrawal. Benzodiazepines were the most mentioned drug class in 32 (62.7%) threads; eight different benzodiazepines were identified including diazepam (16 threads) and chlordiazepoxide (four threads). The next most popular drugs all acted to up-regulate GABA activity: pregabalin (26 threads), phenibut (21 threads) and gabapentin (seven threads). A further 12 drugs were mentioned on a single occasion and these included beta-blockers, antipsychotics and antihistamines. No threads were found to be providing advice on how to obtain medication for GHB withdrawal.

#### Other relevant websites

In addition to forums, the grey literature search identified a number of websites relevant to the use of baclofen in GHB withdrawal. Two clinical protocols for managing GHB/GBL withdrawal were found, one linking to content from ‘The Maudsley Prescribing Guidelines in Psychiatry’ and the other, a document that appeared to originate from another NHS institution, although this could not be verified [51, 52]. Furthermore, four services were identified that offer assistance with GHB/GBL detoxification, of which one was NHS funded and three located in the USA [53–56].

### Baclofen snapshot survey

#### The availability of baclofen

Forty-one unique websites selling baclofen online were identified from a total of 195 search results.47 search results involved the addition of a path (e.g. /buy-baclofen.html) to an unrelated web domain so as to facilitate redirection of the Internet user to an online pharmacy. Of these redirections, a single website was the destination in 26 (55.3%) instances [57]. All re-directed search results were reviewed 1 month later and all of the unrelated paths had been de-activated.

**Fig. 2:**
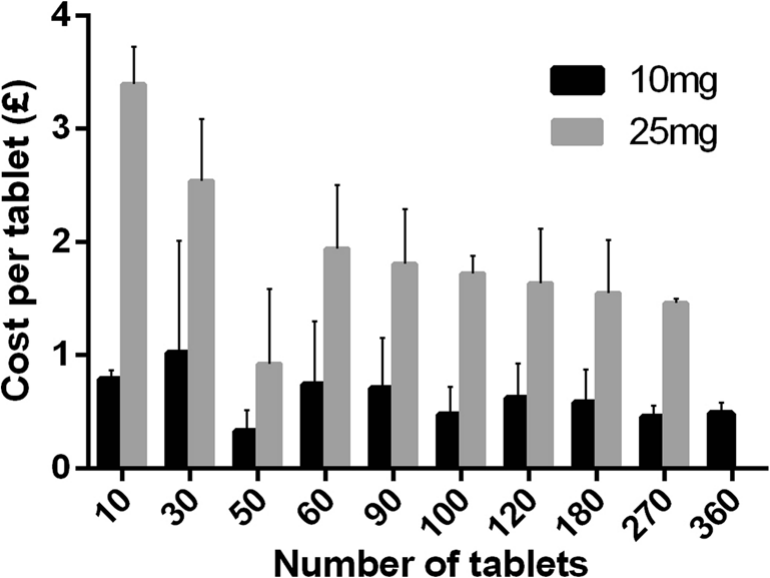
Price of baclofen according to dose and number of tablets purchased. Data expressed as mean ± SD. Tablet quantities available on less than three websites were excluded from this analysis as they may represent non-standard dispensing practices. [SD: standard deviation]

All websites offered baclofen as either tablets or pills, with the exception of a single website offering powder for research use [58]. None of the websites offered ampoules for intrathecal administration. Baclofen was available as either 10 mg tablets (40 (100%) websites), 20 mg tablets (3 (7.5%)) or 25 mg tablets (29 (72.5%)). The advertised purchase price of baclofen generally declined with increasing volume purchased but was not correlated significantly for either 10 mg (Spearman *r* = − 0.55; *p* = 0.10) or 25 mg tablets (*r* = − 0.65; *p* = 0.07) (Fig. 2). For websites offering more than one dose, the mean ± SD cost of baclofen was significantly lower for the 10-mg preparation (£54.47/g ± 17.45) than for the 25-mg preparation (£78.81/g ± 23.56; *p* < 0.01).

#### Website characteristics

Thirty-eight of the 40 websites advertised shipping of baclofen tablets to the UK, with only a single website operating with appropriate regulatory approval. Further information on website characteristics can be found in Supplementary Data.

## Discussion

Since the first case of GHB withdrawal reported in the medical literature in 1994 [7], there has been uncertainty as to the most effective pharmacotherapy to manage this life-threatening condition. High-dose benzodiazepines have become established as the first line approach, probably due to clinician familiarity with their titrated use in alcohol withdrawal. However, as demonstrated in the cases identified, high-dose benzodiazepines are not always sufficient to manage GHB withdrawal [19, 31, 35].

Despite the clinical experience of our centre in which we have successfully treated dozens of patients with GHB/GBL withdrawal with baclofen, there is little published data with regard to the use of baclofen to manage GHB withdrawal. The case reports we identified suggested clinical utility with an acceptable safety profile, but it is not possible to draw definitive conclusions in the absence of randomised controlled trials. Furthermore, despite its described use in both inpatient and outpatient settings, there remains a potential risk of significant harm in baclofen overdose [36, 59].

To augment the limited published literature on the topic, we applied data triangulation techniques on publicly accessible Internet resources to identify sources of ‘grey literature’. We reviewed threads across eight different Internet forums and found multiple mentions of baclofen used to treat GHB withdrawal. The earliest thread identified was published in 2008 and corresponded to the publication of the second case report [19].

The grey literature is at least as extensive as the published scientific literature, with clear caveats relating to accuracy of information and lack of peer review. The dangers of grey literature are clearly demonstrated by one forum post recommending 4 g baclofen to manage withdrawal [47]. The use of baclofen for the management of GHB/GBL withdrawal is off-label, but for other indications, the British National Formulary recommends a starting dose of 5 mg TDS with titration to a maximal daily dose of 100 mg [60]. Doses greater than 200 mg are associated with reduced conscious level, hypotonia and respiratory depression [61], with death having been previously reported after ingestion of 1.25–2.5 g baclofen [59].

The Internet snapshot survey revealed that baclofen is freely available to purchase from online pharmacies that appear to operate contrary to accepted legal and ethical standards. UK/EU registered online pharmacies represent the minority of retailers offering baclofen for sale. It is not possible to determine whether the unregistered online pharmacies are unaware of the legislative requirements or whether they are deliberately contravening the legislation, although a number of factors point towards the latter situation in many instances. The obscuration of both domain ownership and physical location, advertised prescription-free service and the apparent hacking on unrelated websites to generate Internet traffic all suggest deliberate illegal activity. These data are consistent with a report by The Center for Safe Internet Pharmacies which estimates that there are 30–35,000 unregulated online pharmacy websites that are operated by 2–3500 parties [62]. The principle danger associated with these websites is that consumers can self-prescribe from an almost limitless catalogue of medicines without regulatory assurances that these medicines are not counterfeit, unlicensed or withdrawn, and without oversight of the process by a qualified healthcare professional [62, 63].

Despite recent action to suspend UK-based online pharmacies where Care Quality Commission (CQC) inspectors found ‘significant clinical safety and organisational risk to patients’ [64, 65], the current regulatory approach of focusing only on UK-based providers is failing to control online pharmacies whose activities are not restricted by national borders. There is no internationally accepted definition to categorise the illegal selling of medicines, and this combined with the absence of centralised Internet governance has led to fragmented regulation [66]. Until there is a coordinated international approach to both regulation and consumer education, then these websites will continue to put the public at risk of serious harm.

## Conclusions

Despite GHB dependency and withdrawal representing a growing public health challenge, there is a lack of robust clinical data to guide its management. Pre-clinical data combined with a limited number of case reports suggest that the GABA-B agonist baclofen is a useful adjunct to high-dose benzodiazepines, and that its use within a supervised clinical setting is safe. However, Internet forums demonstrate that the use of baclofen to treat GHB withdrawal is not limited to healthcare professionals as self-medication using wide variation in dosage is accepted within the online community. Despite baclofen being a POM, our research into online pharmacies has revealed that it is freely available without prescription and that the majority of online pharmacies identified are non-compliant with UK regulation. This work has highlighted that clinical studies are required into the efficacy of baclofen in the treatment of GHB withdrawal, and that current regulation of online pharmacies is inadequate to protect the general public from harm.

### Statement on contributorship and the guarantor

CNF collected the primary data and undertook the initial data analysis; all authors contributed equally to the drafting of this manuscript. PID acts as guarantor.

## Electronic supplementary material

ESM 1 (DOCX 18 kb)

Supplementary Figure 1 (JPEG 66 kb)

Supplementary Figure 2 (PNG 279 kb)
