# Supplementary material for: Baclofen in gamma-hydroxybutyrate withdrawal: patterns of use and online availability
Source: Eur J Clin Pharmacol. 2017 Dec 3;74(3):349–56. doi: 10.1007/s00228-017-2387-z (PMC5808054; doi:10.1007/s00228-017-2387-z)
Supplement: Supplementary file 1 — (DOCX 18 kb) [file 228_2017_2387_MOESM1_ESM.docx]

**Supplementary Data**

*Website characteristics*

Thirty eight of the 40 websites advertised shipping of baclofen tablets to the UK and further analyses will focus on these with respect to UK regulations. Only a single website had UK registration as assessed by the display of the European Union (EU) Common Logo and registration on the Medicine and Healthcare products Regulatory Agency (MHRA) ‘online medicines seller registry’. A further two online pharmacies provided evidence of professional registration outside of the EU, but this could not be verified.

Evidence of a prescription for prescription-only-medication (POM) was required by ten online pharmacies (26.3%), but only the UK registered pharmacy adhered to the requirement to receive either the original or an electronic copy transmitted using the Electronic Prescription Service (EPS). The other pharmacies requested proof of prescription by the less stringent criteria of receiving a digital copy either by fax or email. There appeared to be no process for confirming that purchaser was indeed the person named on the prescription.

Geographical location of the online pharmacies could be confirmed in only four instances where a physical address was provided; two of pharmacies were located in Canada, one in New Zealand and one in the UK. A further five provided a statement regarding location, in 19 cases the brand identify suggested a country of origin and in eight instances there was no location information provided. A total of seven online pharmacies appeared to be based in the EU, with only the UK registered pharmacy displaying the appropriate EU Common Logo.

The WHOIS queries revealed poor agreement between apparent geographical location of the online pharmacy and the contact address provided by the domain owner. Domain registration was spread across 17 countries (including seven in the EU) and five continents, with country level agreement between physical and digital location occurring in only five instances. Russia demonstrated the greatest location discrepancy as despite no physical locations for online pharmacies, it was the locations of six domain registrations. Figure 3 shows the discrepancy between physical and digital location according to continent.

Furthermore, the WHOIS queries revealed that 20 (52.6%) of domain registrations were held by third party companies who exist to obfuscate ownership. This compared to 7.5% when we performed the same analysis on a non-randomised sample of the MHRA’s online seller database (*n*=40).

*Consumer experience*

The majority of websites provided a consumer experience that included features designed to attract and retain customers. Nine websites (23.7%) contained integrated language translation and 21 (55.3%) enabled medication purchase in multiple currencies. Product information was provided by 25 website (65.8%) and the ability to leave product reviews or website testimonials was advertised in eight (21.1%) instances. Reviews or testimonials were published on four webpages offering baclofen for sale, although none of the comments mentioned baclofen specifically. Only two websites did not appear to provide customer support via the provision of a telephone number, email address or ‘live chat’ facility.

**Supplementary Figure 1:** Summary of search strategy used to identify cases.

**Supplementary Figure 2:** Geographical distribution of advertised website location versus domain registration location.
