# Supplementary figures and images for: Baclofen in gamma-hydroxybutyrate withdrawal: patterns of use and online availability
Source: Eur J Clin Pharmacol. 2017 Dec 3;74(3):349–56. doi: 10.1007/s00228-017-2387-z (PMC5808054; doi:10.1007/s00228-017-2387-z)

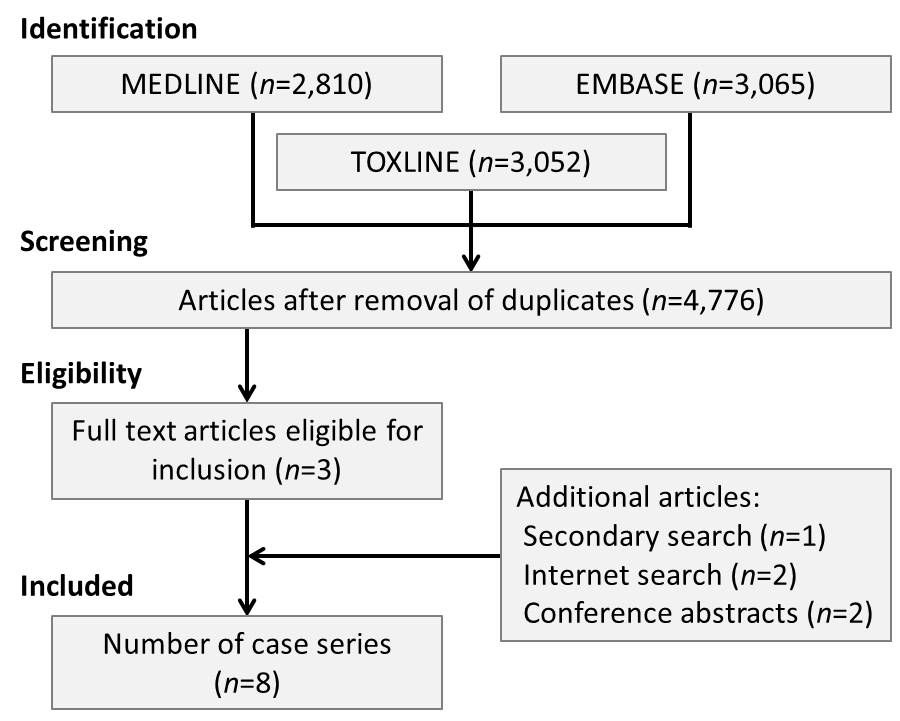

Supplement: Supplementary file 2 — (JPEG 66 kb) [file 228_2017_2387_MOESM2_ESM.jpg]

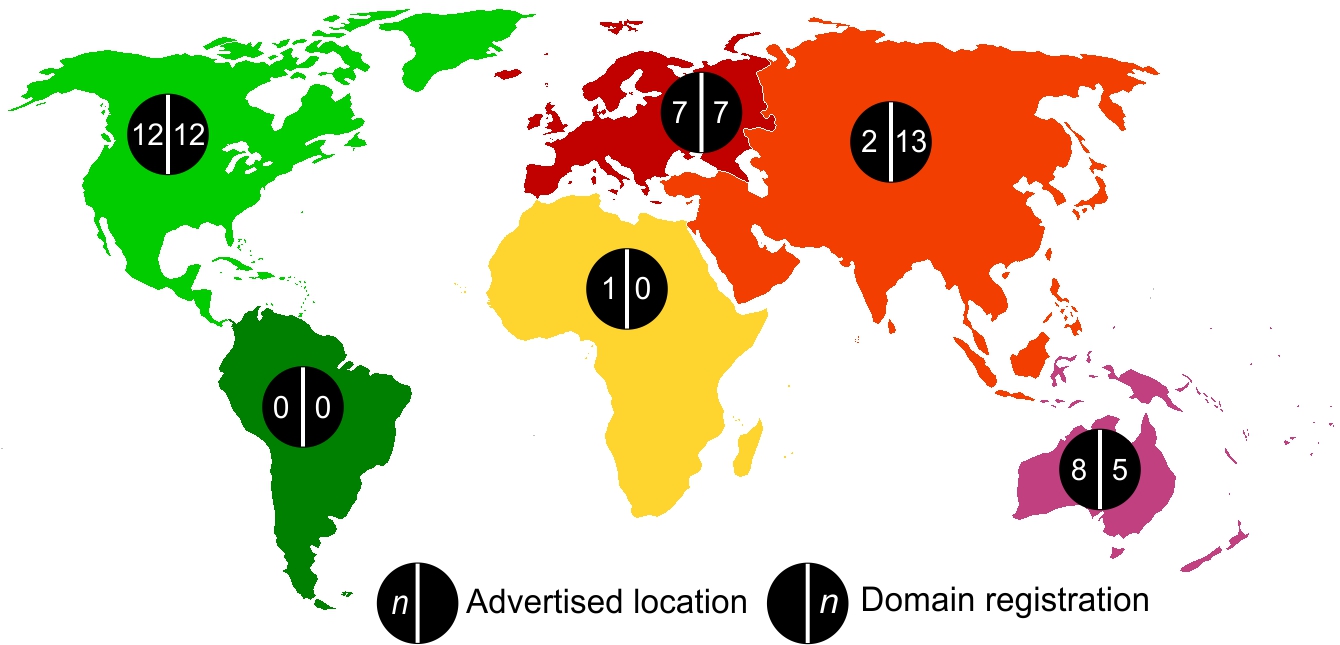

Supplement: Supplementary file 3 — (PNG 279 kb) [file 228_2017_2387_MOESM3_ESM.png]
